# Analysing the Unequal Effects of Positive and Negative Information on the Behaviour of Users of a Taiwanese On-Line Bulletin Board

**DOI:** 10.1371/journal.pone.0137842

**Published:** 2015-09-10

**Authors:** Shu-li Cheng, Wen-hsien Lin, Frederick Kin Hing Phoa, Jing-shiang Hwang, Wei-chung Liu

**Affiliations:** Institute of Statistical Science, Academia Sinica, Taipei, Taiwan; Hangzhou Normal University, CHINA

## Abstract

The impact of social influence causes people to adopt the behaviour of others when interacting with other individuals. The effects of social influence can be direct or indirect. Direct social influence is the result of an individual directly influencing the opinion of another, while indirect social influence is a process taking place when an individual’s opinion and behaviour is affected by the availability of information about others’ actions. Such indirect effect may exhibit a more significant impact in the on-line community because the internet records not only positive but also negative information, for example on-line written text comments. This study focuses on indirect social influence and examines the effect of preceding information on subsequent users’ opinions by fitting statistical models to data collected from an on-line bulletin board. Specifically, the different impacts of information on approval and disapproval comments on subsequent opinions were investigated. Although in an anonymous situation where social influence is assumed to be at minimum, our results demonstrate the tendency of on-line users to adopt both positive and negative information to conform to the neighbouring trend when expressing opinions. Moreover, our results suggest unequal effects of the local approval and disapproval comments in affecting the likelihood of expressing opinions. The impact of neighbouring disapproval densities was stronger than that of neighbouring approval densities on inducing subsequent disapproval relative to approval comments. However, our results suggest no effects of global social influence on subsequent opinion expression.

## Introduction

People tend to adopt the behaviour of others or change their thoughts or attitudes when interacting with other individuals [1]. This is the process of social influence. Such influence is likely to occur especially when people face uncertain situations. The reasons that individuals acting in accordance with others’ behaviour may be that, for example, they believe others are better informed [2] or in order to avoid being seen as a deviant [3]. The effects of social influence can be *direct* or *indirect* [4]. The former is the result of an individual directly influencing the opinion of another, typically by persuasion or coercion [4] and is also the usual focus of studies of social influence [2]. The latter is a process taking place when an individual’s opinion and behaviour is affected by the availability of information about others’ actions [4]. Although direct effects of social influence are important, indirect effects are not negligible. This is especially true in the on-line environment where network and digitisation technologies have reduced the cost of information production and dissemination [5].

As more people participate in an event or activity, more information is gathered through their actions and consequently the impact of indirect social influence becomes more important. An individual who takes action subsequently therefore has the benefits of evaluating the consequence of others’ behaviour in order to avoid adverse outcomes. Such conformity is associated with the size of the reference group [6] or the proportion of others who have already taken action [7]. This collective behaviour is frequently discussed in the context of individual thresholds [7–10]. Here a threshold refers to the minimum number (or proportion) of other individuals a person would like to see involved before he would take action [8]. Some individuals may participate in an activity after few others have done so whilst some may wait until almost everyone has joined in. Therefore, the diffusion rate of an event depends upon the distribution of individual thresholds. Low thresholds increase the likelihood of an event taking place throughout the population in a short time whereas high thresholds hinder the occurrence of an event.

Social influence on collective behaviour may originate from different sources [11,12]. What has been discussed so far implies individuals have a *global* vision of information on others’ (collective) behaviour. Although global information is undoubtedly important, individuals may not always have an accurate perception of the whole community when making their decisions and as such they may prefer to rely on information from their *local* network such as friends or acquaintances [12–14] or, in general, socially proximate others [15]. In this case, an individual may take into account the decisions made by his close peers or simply follow the most prevalent trend at that time point, and adopts the most appropriate course of action.

Although the study of social influences on one’s behaviour has long been a research topic in sociology, they are mostly limited to situations found in real societies. With the advent of internet and ever-improving computing technology, people nowadays spend a substantial amount of time on-line for work and leisure purposes. It is therefore of great interest to understand how social influences operate in the on-line community. Off-line and on-line communities also differ in how information is presented to people. For instance, traditional research on collective action [7,8,10,16] mostly focuses on the impact of individuals who have made the same decision and is therefore likely to ignore alternative options or opposite opinions. This often happens in the off-line world when people may not have information on alternative choices. However, this can change with the use of computer technology and the internet as information of all sorts are recorded in written form providing a much more diverse view to people [17]. Hence in the on-line community, people have the advantage and opportunities to evaluate such a heterogeneous set of information before making their decisions. Indeed, Muchnik, et al. [18] adopted an experimental approach to study how pre-existing on-line ratings affect subsequent users’ judgments, and found that people were heavily influenced by the opinions which other people expressed on-line. In other words, on-line ratings were found to exhibit the likes-bred-likes phenomenon. Herding behaviour was evident among their commenters. Moreover, Muchnik, et al. [18] also observed certain asymmetry in herding effects for positive and negative social influences with the former being more likely to create opinion herding than the latter.

Given that the study of social influences in the on-line community is still in its infancy and data on this regard are becoming increasingly available, we argue that there is a need to understand how people act when facing uncertainties in the on-line community. This paper seeks to examine how previously expressed opinions influence individuals’ subsequent choices of action through the mechanism of indirect social influence in the on-line community. We do not attempt to elucidate the incentive of people’s action in participating in discussion forums, but instead we focus on whether social influence is at work from the global or local levels. Specifically, we use data from an on-line bulletin board to examine the effects of positive and negative information on individuals’ choices of opinion expression (i.e., approval or disapproval comments) and in what way this is achieved (i.e., globally or locally). A parsimonious model is used to answer these questions. The results have provided a good indication on how indirect social influence affects individuals’ decisions.

## Methods

### PTT data

This manuscript studies the behaviour of anonymous individuals in an electronic bulletin board community, and the data were analyzed anonymously. We did not conduct experiments on human participants or manipulate the bulletin board in anyway to affect users' behavior. We simply analysed their actions by using data that are open and free to the general public. The data only contained usernames, and not any information regarding the users’ real identity or IP addresses. The findings from this study were not related to or did not identify any specific individuals. We made an informal inquiry about whether our study needed IRB review and the institutional review board of Academia Sinica (Taiwan) made clear to us that because we did not collect or manipulate experiment on human subjects, no IRB review was required.

The data used in the following analysis were collected from the PTT Bulletin Board System (PTT for short) in Taiwan. This bulletin board system allows user access via a telnet protocol and provides an instantaneous and free of charge online forum community. The site was originally founded at the National Taiwan University in 1995 [19]. By the year 2000 the population of registered users had grown rapidly, and it has become the largest online discussion forum in Taiwan [20]. Although bulletin board systems rapidly faded in popularity after the use of the internet became widespread in the 1990s, PTT bulletin boards system remains an extremely popular form of communication among Taiwanese youth [20]. On average, there are about a million users on PTT everyday [20].

In the PTT, the registered users choose their usernames when creating accounts. Usernames are chosen in such a way that users’ identities cannot be recognised. Therefore, even though usernames are shown when users post messages or comments on others’ messages, their true identities are also protected. Consequently, the data used in the analysis will be treated as if all users are anonymous.

**Fig 1:**
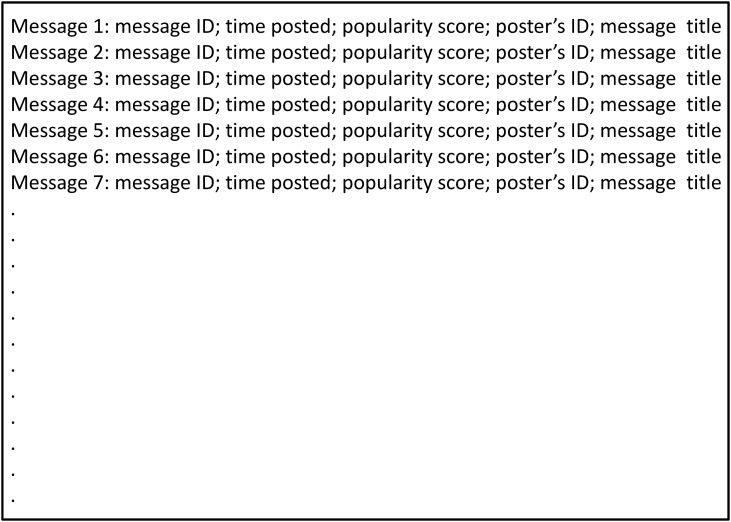
PTT user’s interface. A schematic representation showing a typical PTT board on a user’s interface. The posted messages are arranged in chorological order.

**Fig 2:**
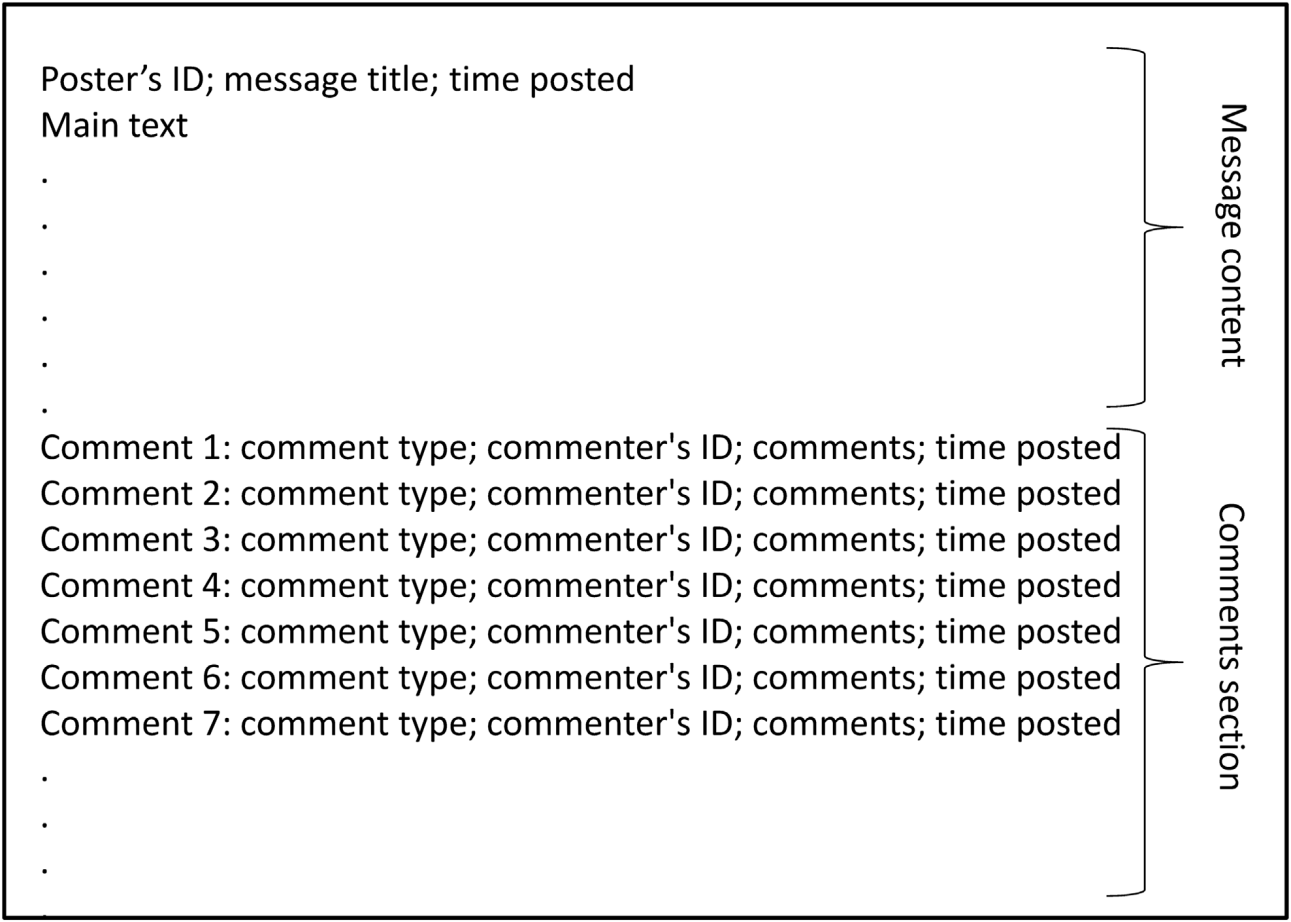
An illustration of a posted message with comments. A schematic representation showing a series of comments attached to a posted message in a PTT board. The comments are recorded in the order they appear.

Once logged on to the PTT, users can perform functions such as posting, reading and commenting messages. The communication forum is organised by classifications, for example, sports and movies, etc. Under each classification, there are several topic boards serving as platforms for people to participate in various discussions. For example, under ‘sports’ classification, there are boards like American Major League Baseball and World Cup Football. A board presented on a user’s interface is depicted in Fig 1. Here, in chronological order, users are presented with a list of messages created by other users. Each row of the interface represents a message and summarises basic information about this message, these include: message identification number, popularity score (see below), the username of the person who created this message, and the main title of the message. In a board, the users can read messages and comments as well as posting their own ones. In addition, they can comment on other users’ messages by using three pre-classified categories, namely approval, disapproval or neutral. The users choose which category can best describe their comments towards the message and all these comments are recorded chronologically. In other words, the data are presented in such a way that each message contains a series of comments in the order they appeared. Fig 2 shows how this may look like in the PTT. Thus, a user has the opportunity to read the message and all previous comments before he posts his own comments. The difference between the numbers of approval and disapproval comments is the popularity score of the corresponding message, and this score is presented on the board page when a user browse through the list of current messages.

Among all boards in PTT, the gossiping board is the most popular and attracts the most users almost every day. Although it was initially created to provide gossips on celebrity stories, now the board contains a variety of topics including for example, politics, media or live news [19]. It attracts about twelve thousands users simultaneously in a popular period and thousands of them usually respond by writing comments to posted messages. Moreover, comments in the gossiping board usually appear instantaneously. The popularity of the gossiping board in Taiwan and its wide range of topics covered, as well as the instantaneous actions of its users, are the reasons why we chose the gossiping board for analysis in this paper.

### Data selection criteria

As mentioned in Introduction, this paper investigates the impact of previously expressed comments (therefore opinions) on subsequent users’ behaviour. Specifically, we are interested in the extent to which a user’s choice of approval or disapproval towards a posted message was affected by (or dependent on) the action of previous users. Each approval or disapproval comment was considered as a microscopic social stimulus for later users. To make comparisons between approval and disapproval comments reliable and meaningful, the data should have the possibility of reflecting both types of opinions and should include a wide range of subjects. Given that the gossiping board contained a wide range of topics and variety of users, we further set some criteria on how data were selected for analysis. Firstly, we restricted our data to those messages containing a proportion of approval comments between 40 and 60 per cent so that both approval and disapproval opinions were likely to be present. Secondly, in order to avoid short series of comments, the messages selected for analysis contained at least 100 comments. Because of data availability, we used the messages from the gossiping board from 6^th^ March 2007 to 5^th^ February 2010 for our analysis. The final sample used in our analysis consisted of 56 messages and there were about 20,000 comments in total.

### Model

A statistical model was constructed to examine the relationship between social influence and opinion expressions in the PTT gossiping board. Here, opinion expressions are the types of comments expressed by users for posted messages. They are also the response variable in the model. As mentioned earlier, the response variable has three categories, i.e. approval, disapproval and neutral. However, neutral comments in our data deserve some further description. Unlike approval or disapproval comments, neutral comments may arise in three ways. Firstly, the group includes users’ true neutral comments. Secondly, if a user’s comment takes more than one line to complete, then the system will automatically mark those extra lines as neutral regardless of his opinion expressed in the first line. Finally, the comments expressed by the same user within five minutes of previously expressed comments are also marked as neutral. In other words, the neutral category does not truly identify neutral comments, and it consequently cannot be considered as neutral comments as one would generally expect. Therefore, we decided not to analyse neutral comments in the model. Since there are only two categories considered in this study, logistic regression analysis was then employed here [21]. The logistic model estimates the log-odds for one category relative to the baseline category as a function of the explanatory variables. Here approval comments are chosen as the baseline category and the measures of social influence are the explanatory variables.

We distinguish two sources of social influences in the PTT community when a user reads a posted message. The first type is the *global* social influence and we assume a user is exposed to such an influence when he sees the popularity score of a posted message while browsing the board page. As mentioned earlier, the popularity score is the current difference between the numbers of approval and disapproval comments, indicating the overall trend of comment types for a posted message. The popularity score as a variable measuring global social influence is akin to threshold models of collective behaviour [8,22], and it takes every unit of difference in approval and disapproval comments as a stimulus for later users’ opinion expression. Furthermore, this allows us to examine the unequal effects of approval and disapproval comments on subsequent users’ behaviour. We also assume a user can be exposed to local social influence. Following a posted message, a user is also presented with comments from other users; and we define local social influence as the trend exhibited in the most recent (or neighbouring) comments. Since there are around 25 comments on the display window of a user’s interface, therefore the most recent comments consist of those 25 comments right before a user posts his own comment. However, since the number of comments displayed on the user’s interface window depends on factors such as font size, we also analysed cases with other numbers of comments in order to check the robustness of our findings. Specifically, we considered numbers of comments from five to fifty with increment steps of five.

We assume local social influence consists of two variables measuring respectively the densities of approval and disapproval comments among the neighbouring opinions right before a user posts his comments. Having two density measures for local influence allow us to evaluate and compare the clustering effects of approval and disapproval comments on later users’ behaviour. Whether comments of the same type, the opposite type, or both types have an important role in influencing later opinions can be evaluated.

The equation of logistic regression model for disapproval comment relative to approval comment is:

$$\text{log}\left(\text{Pr}(Y_{it}=D)/\text{Pr}(Y_{it}=A)\right)=\alpha +\beta_{1D}X_{1}+\beta_{2D}X_{2}+\beta_{3D}X_{3}$$ (1)

where *A* and *D* respectively denote approval and disapproval comments, and taking *k* = 25 as the most common norm, then

$$X_{1}=\sum_{j=1}^{i-1}I(y_{jt}=A)-\sum_{j=1}^{i-1}I(y_{jt}=D)$$ (2)

$$X_{2}=\sum_{j=i-k}^{i-1}\left(I(y_{jt}=A)/k\right)\text{, where }k=25\text{ and }i>k$$ (3)

$$X_{3}=\sum_{j=i-k}^{i-1}(I(y_{jt}=D)/k)\text{, where }k=25\text{ and }i>k$$ (4)

where *I*(*expression*) is a function which evaluates to 1 if the expression in the parenthesis is true. In Eq (1), random variable *Y*_*it*_ denotes the type of the *i*-th comment in message *t*, which can either be an approval comment (*A*) or a disapproval comment (*D*). *X*_1_ represents the global measure up to the (*i*-1)-th comment in message *t*. It is the difference between the amounts of approval and disapproval comments taking place before the current one. *X*_2_ and *X*_3_ represent the neighbouring (or local) densities of approval and disapproval comments respectively among the 25 nearest comments before the current one. *α* and *β*s are the coefficients to be estimated.

In summary, the model seeks to test the following hypotheses:

*H*_0_: There is no relationship, meaning *β*_1D_ = *β*_2D_ = *β*_3D_ = 0, between the log odds of expressing a disapproval comment relative to an approval comment and the effects of global and local social influences.
*H*_*a*_: There is a relationship.

### Model prediction

Next we assess the prediction accuracy of our statistical model. However, obtaining future series of comments of the 56 messages in the analysis to evaluate predicted outcomes is not possible. We therefore propose to use the last 20 comments of each message as the test data. In other words, the model will be refitted using the original data without the part of the test data. After obtaining the new parameter estimates, we generate the next 20 predicted outcomes for each message and compare them to the test data; and we then calculate the accuracy of our prediction.

### Analysis using randomised empirical data

Empirical data analysis in social studies often lack of control groups to validate its findings. Therefore, many studies that evaluated the impact of social influence adopted an experimental approach to overcome this problem, for example, Salganik, et al. [23] and Mavrodiev, et al. [4]. However, such an experimental approach was not a possible option at the time when our data were collected. In order to serve as a control to our analysis here, we randomised the order of the observed comments within each message and performed the same analysis on the randomised data many times. We then treated those results as baseline results of the control group and compared them to the results obtained using empirical data.

## Results

### Fitting statistical model to empirical data

Table 1 shows the estimated logistic regression coefficients for the fitted model. We see that the impact of global social influence on the log odds of disapproval relative to approval is not statistically significant when controlling for local social influence. In contrast, the effects of local approval and disapproval densities are significant on the log odds when allowing for global social influence. In a nutshell, the results suggest that there is a relationship between local social influence and the log-odds of expressing disapproval relative to approval comments.

**Table 1:** Estimated parameter values of the logistic regression model

	Estimate	SE
*α* [intercept]	-0.262	0.072
*β*_1D_ [global]	0.000	0.000
*β*_2D_ [local—approval]	-1.973***	0.127
*β*_3D_ [local—disapproval]	2.696***	0.110

Note: significance:

***p-value<0.001.

If the local approval proportion is to increase by one point, then the log odds of expressing disapproval relative to approval will be expected to decrease by 1.96 units while holding all other variables in the model constant. In other words, if the proportion of approval comments is currently increasing, a subsequent user is less likely to express disapproval comments. Furthermore, the relationship between the log odds of expressing disapproval relative to approval comments and local disapproval densities is positive. A one-unit increase in neighbouring disapproval proportions is associated with 2.67 units increase in the relative log odds of expressing disapproval versus approval comments. All these suggest that users are likely to express the comment type that is most prevalent in the preceding neighbouring comments. Note that the unequal effects of local densities of approval and disapproval comments are discernible. Disapproval comments are more likely to bring out comments of the same type than their approval counterparts.

Table 2 shows the results of estimated parameters for different values of *k*, starting from five to fifty in steps of five (apart from *k* = 25 since the results are presented in Table 1). We see that the impact of global social influence remains insignificant. In contrast, the impact of local social influence is statistically significant in all models. This supports the results in Table 1. Hence, the effects of local approval and disapproval densities in influencing subsequent comment expressions seem reliable. Moreover, the coefficients for local approval and disapproval densities are becoming larger in absolute terms as *k* increases. A bigger *k* implies more local information is available. Therefore, this suggests as more local information is available, its impact on comment expressions also increases.

**Table 2:** Estimated parameter values for *k* = 5 to *k* = 50 in steps of 5 (apart from *k* = 25).

	*Disapproval*	Estimate	SE
K = 5	*α* [intercept]	-0.273	0.054
	*β*_1D_ [global]	-0.001	0.000
	*β*_2D_ [local—approval]	-0.935***	0.085
	*β*_3D_ [local—disapproval]	1.672***	0.080
K = 10	*α* [intercept]	-0.309	0.064
	*β*_1D_ [global]	0.000	0.000
	*β*_2D_ [local—approval]	-1.398***	0.105
	*β*_3D_ [local—disapproval]	2.232***	0.095
K = 15	*α* [intercept]	-0.305	0.068
	*β*_1D_ [global]	0.000	0.000
	*β*_2D_ [local—approval]	-1.633***	0.115
	*β*_3D_ [local—disapproval]	2.461***	0.102
K = 20	*α* [intercept]	-0.281	0.071
	*β*_1D_ [global]	0.000	0.000
	*β*_2D_ [local—approval]	-1.826***	0.122
	*β*_3D_ [local—disapproval]	2.592***	0.107
K = 30	*α* [intercept]	-0.262	0.074
	*β*_1D_ [global]	0.000	0.000
	*β*_2D_ [local—approval]	-2.031***	0.130
	*β*_3D_ [local—disapproval]	2.772***	0.113
K = 35	*α* [intercept]	-0.231	0.074
	*β*_1D_ [global]	0.001	0.000
	*β*_2D_ [local—approval]	-2.126***	0.133
	*β*_3D_ [local—disapproval]	2.797***	0.114
K = 40	*α* [intercept]	-0.222	0.075
	*β*_1D_ [global]	0.001	0.000
	*β*_2D_ [local—approval]	-2.156***	0.135
	*β*_3D_ [local—disapproval]	2.822***	0.116
K = 45	*α* [intercept]	-0.219	0.076
	*β*_1D_ [global]	0.001	0.000
	*β*_2D_ [local—approval]	-2.179***	0.137
	*β*_3D_ [local—disapproval]	2.859***	0.117
K = 50	*α* [intercept]	-0.216	0.076
	*β*_1D_ [global]	0.001	0.000
	*β*_2D_ [local—approval]	-2.190***	0.138
	*β*_3D_ [local—disapproval]	2.882***	0.119

Note: significance:

***p-value<0.001.

### Results for model prediction

Table 3 shows the estimated logistic regression coefficients for the refitted model without the test data. We see that the results are close to those of using all comments. That is, the impact of global social influence is not statistically significant on the log odds when controlling for local social influence. However, there is still a relationship between local social influence and the log-odds of expressing disapproval relative to approval comments. The overall accuracy of the prediction is 0.62 (see Table 4).

**Table 3:** Estimated parameter values of the logistic regression model (without test data).

	Estimate	SE
*α* [intercept]	-0.302	0.076
*β*_1D_ [global]	0.000	0.000
*β*_2D_ [local—approval]	-1.972***	0.133
*β*_3D_ [local—disapproval]	2.776***	0.118

Note: significance:

***p-value<0.001

**Table 4:** Accuracy of test data prediction based on the proposed logistic regression model

	Approval observed	Disapproval observed
Approval predicted	186	179
Disapproval predicted	247	508

The accuracy is 0.62 = (186+508)/(186+179+247+508).

### Results from randomisation

We repeated our analysis on 30 randomised datasets and Table 5 shows the result from one such analysis. We see that, unlike the results of the original data, it is only the impact of global social influence that is significant after randomising the orders of the comments. The reversed statistical significance of global and local social influence provides further evidence that there is a strong relationship among the neighbouring comments in the PTT data, suggesting the clustering effect of user behaviour.

**Table 5:** Estimated parameter values for the randomised data

	Estimate	SE
*α* [intercept]	0.039	0.077
*β*_1D_ [global]	-0.003***	0.000
*β*_2D_ [local—approval]	-0.218	0.139
*β*_3D_ [local—disapproval]	0.007	0.149

Note: significance:

***p-value<0.001.

## Discussion

### Impact of social influence

In this paper, we examined the dependence of users’ behaviour of a popular electronic bulletin board and empirically quantified the impacts of two different types of social influence on users’ choices of expressing approval and disapproval towards posted messages. By analysing the data from the PTT gossiping board, our results suggest that users’ choices of opinion expression depend positively on the proportion of previous users who have expressed the same idea and negatively on the proportion of the opposite opinions in the neighbouring preceding comments. The results give evidence that, although in an anonymous on-line community, social influence still plays an important role in affecting users’ behaviour. When expressing an opinion, individuals are sensitive to what has happened around them and the magnitude of it. The fact that a user tends to take into account of what neighbouring others are doing before taking his own action, is similar to the findings in named situations from the literature [7–9,24].

Our findings suggest that when controlling for both global and local social influences, the significant effect, in fact, comes from local social influence. A user’s behaviour is more likely to be affected by what is the most popular opinion at the time shortly before he takes action. On this regard, we have found that decisions made by neighbouring others (or the current popular trend) can have a strong impact on subsequent users’ behaviour. Such important local effects from socially proximate others are also found in the literature [10,15,25]. Here, our results show that users tend to go with what is the most fashionable or popular thing to do at the very moment in time resulting in the emergence of clusters of similar opinions. This is sensible as, intuitively, doing things as what others have been doing reduces the risks of many possibilities. When facing uncertainty, other people’s decisions can serve as useful information for a person [26]. For risk-averse individuals, this will reduce the cost of uncertainty.

### The unequal effects of local social influences

Increasing local approval proportion tends to decrease the relative log odds of expressing disapproval versus approval comments for subsequent users, whereas increasing local disapproval proportion shows the opposite effect. Moreover, the magnitudes of these effects are not similar. Our results show that the effect of disapproval comments is stronger than that of approval comments. This finding is in line with studies on the positive-negative asymmetry in information processing where negative information is found to affect an individual’s behaviour more strongly than positive information [27–30]. Dong, et al. [31] pointed out that the reason for such asymmetry may be due to human brain being wired to pay more attention to negative rather than positive experiences or information. In other words, our brains are more actively looking for negative information instead of positive ones.

### Anonymity

Anonymity in theory is likely to reduce the intensity of face-to-face interaction among individuals and therefore reduces the impact of social influence from one to another [32]. A recent study in educational technology [33] also pointed out that when anonymous posting was enabled, students were significantly more likely to post to on-line student discussion boards. Anonymity indeed contributes to the rising participation of the on-line community.

Despite increasing participation, we would expect, in the light of PTT being an anonymous environment, that its users’ responses to posted messages should not be affected by earlier comments from other users. However, our results demonstrate this is indeed not the case. Thus our findings join others in the literature (although under a different context) in claiming that anonymity does not lend support to the reduction of social influence [34]. The reason being that anonymity could lead to deindividuation such that the PTT users may identify themselves as a part of the PTT community [35]. The theory suggests that once group identity has been established, social influence can then spread from one member to another [32].

### Prediction accuracy of fitted statistical model

The prediction accuracy of our model here is 0.62. Our model here is of simplistic nature and the focus here is to examine the existence and quantify the effect of global and local social influences. Other factors must be considered in the model in order to predict future comment types to a higher degree of accuracy. For instance, an individual who has expressed a particular comment type toward a posted message might incline to expressing the same type of comment in future. The effect of users’ past behavior can be easily examined here. We fitted a new logistic regression model and found that the effects of users’ past behaviour and local influence are statistically significant, and the model prediction is also improved to 0.69 (see S1 Appendix). Other factors are more difficult to come by with our current data. For instance, a user might have already formed an opinion toward a posted message regardless other users’ comments. Assessing such personal characteristics will pose a new challenge and new data collection is required.

## Conclusions

Using the PTT gossiping data, we have found some interesting findings for the effect of social influence on users’ decisions on showing approval or disapproval towards posted messages. We see that as long as decisions are made in a sequence and observable, individuals tend to use this information to help them make their choices. Our results suggest that such influence occurs at the local level, implying on-line users tend to adopt such information to conform to the majority of the nearby individuals. Moreover, when more local information is available, its impact also increases. Interestingly, even in an anonymous environment where individuals are unknown to each other, there is still a tendency for everyone to follow the present popular trend. If previous information is not revealed to users such that they have little idea on what others have been doing, then whether social influence still plays a role in users’ behaviour in an on-line community remains an elusive question and experiments need to be conducted to investigate this issue. Moreover, the global and local measures in our models assumed independent effects and each comment has an equal weight. In reality, some users may be perceived to be more important (or more prestigious) than others in a community, and their comments may consequently influence others more strongly. All these can be interesting questions and give directions to future research on this particular topic.

## Supporting Information

S1 Appendix NewModel.doc Logistic regression model with users’ past behaviour. In this appendix we formulate the new model and summarise the results on parameter estimates and model prediction.
(DOC)

S1 File Comment sequences analysed in the study. PTT Comments analysed in this study are pooled in one single text file. The headers are: Message_id, the identifier number of a message; Comment_user_id, the identifier number of the user who wrote the comment; Comment_order, this number identifies the chorological order in which a comment appears in the corresponding message; Type_of comment, this identifies the type of the corresponding comment.
(TXT)
