# Supplementary material for: Analysing the Unequal Effects of Positive and Negative Information on the Behaviour of Users of a Taiwanese On-Line Bulletin Board
Source: PLoS One. 2015 Sep 10;10(9):e0137842. doi: 10.1371/journal.pone.0137842 (PMC4565676; doi:10.1371/journal.pone.0137842)
Supplement: S1 Appendix — In this appendix we formulate the new model and summarise the results on parameter estimates and model prediction. (DOC) [file pone.0137842.s001.doc]

**Logistic regression model with users’ past behaviour.** Our current model only consists of predictors for global and local influences. In this new model we further include covariates related to individuals’ past behaviour. In this new model we include two indicator variables associated with users’ past behaviour:

$\log\left(\Pr(Y_{it}=D)/\Pr(Y_{it}=A)\right)=\alpha+\beta_{1D}X_{1}+\beta_{2D}X_{2}+\beta_{3D}X_{3}+\gamma_{1}Z_{1}+\gamma_{2}Z_{2}$.

All terms are as defined as before in the main text with the following exceptions. $Z_{1}=1$ if the individual who made the *i*-th comment had also made approval comments in the *t*-th message before, and 0 otherwise. The other indicator $Z_{2}=1$ if the individual who made the *i*-th comment had also made disapproval comments in the *t*-th message before. γs are the parameters that need to be estimated. We then fitted the new model without the test data, and summarised the result in the following table:

**Table S1A Estimated parameter values of the new model with additional two predictors (without test data)**

|  | Estimate | SE |
| --- | --- | --- |
| *α* [intercept] | -0.530 | 0.088 |
| *β*_1_*_D_* [global] | 0.000 | 0.000 |
| *β*_2_*_D_* [local – approval] | -1.677*** | 0.149 |
| *β*_3_*_D_* [local – disapproval] | 2.786*** | 0.135 |
| *γ*_1_[more preceding approval comments] | -1.667*** | 0.069 |
| *γ*_2_ [more preceding disapproval comments] | 1.812*** | 0.059 |

Note: significance: ****p*-value<0.001

The result shows that these two behavior indicators are very significant in addition to the effect of local social influence. Furthermore the accuracy of the new model is improved to 0.69 as summarised in the following table:

**Table S1B Accuracy of test data prediction based on the new model with additional two predictors**

|  | Approval observed | Disapproval observed |
| --- | --- | --- |
| Approval predicted | 244 | 155 |
| Disapproval predicted | 189 | 532 |

The accuracy is 0.69= (244+532)/(244+155+189+532).
